# Supplementary material for: Effect of social and digital media mental health messaging on mental health help-seeking behaviors in the sub-Saharan African population: A systematic review protocol
Source: PLoS One. 2026 Mar 10;21(3):e0342264. doi: 10.1371/journal.pone.0342264 (PMC12974885; doi:10.1371/journal.pone.0342264)
Supplement: Attachment — Submitted filename: RESPONSE LETTER_PAB_10_09_2025.docx [file pone.0342264.s003.docx]

October 22, 2025

Promila Bishnoi

Editorial Office

PLOS ONE

**Re: Point-by-Point Response to Editorial Decision on Manuscript PONE-D-25-53513**

**“Effect of social media mental health messaging on mental help-seeking behaviors in the sub-Saharan African population: a systematic review protocol”**

Dear Ms. Bishnoi,

Thank you for your editorial decision letter dated October 9, 2025. We greatly appreciate the opportunity to revise and resubmit our systematic review protocol. Below, we provide a detailed point-by-point response to the concerns raised.

**Editorial Concern**: **Language Restrictions Without Adequate Justification**

**Editor’s Comment:**

“We note that language restrictions are imposed on the literature searches without adequate justification. There is increasing recognition that exclusion of studies published in other languages can increase the risk of bias in systematic reviews. We believe that scientific reasons rather than resource constraints should justify any restrictions. As a result, we have concerns about the comprehensiveness of the review and the validity of the conclusions.”

**Our Response:**

We completely agree with this concern and have removed all language restrictions from our protocol. Below are the specific changes made throughout the manuscript:

**Change 1 - Abstract (Page 1)**

Original: “...will be systematically queried using predefined keywords.”

Revised: “...will be systematically queried using predefined keywords without language restrictions to ensure comprehensive evidence capture.”

**Change 2 - Search terms, and Search Strategy for identifying studies Section (Page 11)**

Original: “Only studies in English will be included.”

Revised: “Without language restriction, we will conduct an extensive search for primary studies published between January 2000 and September 2025, a period that reflects the rise and widespread use of social media.”

**Change 3 - Study Selection Section (Page 13) - New Text Added**

Added: “For non-English studies, initial screening of titles and abstracts will be conducted using verified machine translation tools (e.g., Google Translate, DeepL), with cross-verification by bilingual team members where available.”

**Change 4 - Data Extraction Section (Page 14) - New Text Added**

Added: “For studies published in languages other than English, data will be extracted from full-text articles that have either been translated using language translation software or translated by bilingual team members with expertise in the subject. A second reviewer will verify the accuracy of the translations whenever possible, and any uncertainties will be documented and, if needed, clarified with the study authors.”

**Change 5 - Discussion Section (Page 15) - New Text Added**

Added: “A key methodological strength of this review is the absence of language restrictions, which enhances comprehensiveness and reduces selection bias. Given the linguistic diversity of sub-Saharan Africa, this inclusive approach ensures that evidence from Francophone, Lusophone, Arabic-speaking, and other linguistic contexts is not excluded from the synthesis.”

**Rationale For Removing Language Restrictions**

1. Linguistic Diversity in SSA: Research in sub-Saharan Africa is published in English, French, Portuguese, Arabic, Swahili, and other regional languages. Excluding non-English studies would disproportionately exclude evidence from Francophone (Senegal, Côte d'Ivoire, Burkina Faso), Lusophone (Angola, Mozambique), and Arabic-speaking regions (Sudan, Mauritania).
2. Reduction of Selection Bias: Studies in non-English languages may report different findings, and their exclusion can systematically bias systematic review conclusions.
3. Best Practice Alignment: Cochrane and Joanna Briggs Institute guidelines recommend avoiding language restrictions unless scientifically justified.
4. Feasibility: Professional translation services, machine translation with verification, and our diverse team make multilingual inclusion feasible.
5. Comprehensive Evidence: This ensures our findings represent the most complete evidence base for culturally relevant SSA interventions.

**Additional Improvements Made**

1. Corrected inconsistencies where language restrictions were stated as “English only” vs. “English or French” in the original submission
2. Made translation methodology explicit, reproducible, and systematic throughout
3. Enhanced Discussion to acknowledge how this strengthens validity in SSA’s linguistic context
4. Corrected minor formatting issues (extra commas, spacing)

**Conclusion**

These revisions comprehensively address the editorial concerns and align our protocol with PLOS ONE’s standards for systematic reviews. We now provide:

- A clear, defined research question
- A systematic, comprehensive literature review without language-based selection bias
- Clearly reported, reproducible, systematic methods, including translation protocols
- Cultural sensitivity appropriate for SSA’s linguistic diversity

We appreciate this opportunity to strengthen our protocol. The editorial feedback has resulted in a more rigorous, comprehensive, systematic review that will contribute valuable evidence to mental health communication in sub-Saharan Africa.

Thank you.

Sincerely,

Dr. Laura Marciano

Corresponding Author

The Media School, Indiana University Bloomington

Email: laumarci@iu.edu

Telephone: +18128560422

On behalf of all co-authors:

Priscilla Aboagyewaa Boateng, Isaiah Osei Duah Junior, Josephine Ampong, Margaret Dowuona-Hammond, Peter J. Schulz
