# Supplementary material for: Effect of social and digital media mental health messaging on mental health help-seeking behaviors in the sub-Saharan African population: A systematic review protocol
Source: PLoS One. 2026 Mar 10;21(3):e0342264. doi: 10.1371/journal.pone.0342264 (PMC12974885; doi:10.1371/journal.pone.0342264)
Supplement: Attachment — Submitted filename: Reviewers Comments for PONE-D-25-55509.docx [file pone.0342264.s002.docx]

Reviewers Comments for PONE-D-25-55509

1. Does the manuscript provide a valid rationale for the proposed study, with clearly identified and justified research questions? YES
2. Is the protocol technically sound and planned in a manner that will lead to a meaningful outcome and allow testing the stated hypotheses? PARTLY
3. Is the methodology feasible and described in sufficient detail to allow the work to be replicable? YES
4. Have the authors described where all data underlying the findings will be made available when the study is complete? NO, this was not specifically mentioned
5. Is the manuscript presented in an intelligible fashion and written in standard English? NO
6. Review Comments to the Author
   Thank you for the opportunity to review this submission. The paper is well written in clear language. I have noted a few issues to consider detailed below
   a. Data synthesis: The authors stated that synthesis will be structured around population characteristics, intervention characteristics including platform, messaging type, duration, and framing, comparator type, and mental health outcomes including knowledge, attitudes, behaviors, empowerment, and help-seeking. Given the expected diversity of the mental health outcomes probably measured using various tools, how will this be synthesized?
   b. Data availability after the study: This was not explicitly stated in the protocol
   c. Title of the study: Slightly ambiguous, mental help-seeking behaviors, would mental health help seeking behaviors read better?
